# Differential regulation of insulin signalling by monomeric and oligomeric amyloid beta-peptide

**DOI:** 10.1093/braincomms/fcac243

**Published:** 2022-09-24

**Authors:** Rubén Molina-Fernández, Pol Picón-Pagès, Alejandro Barranco-Almohalla, Giulia Crepin, Víctor Herrera-Fernández, Anna García-Elías, Hugo Fanlo-Ucar, Xavier Fernàndez-Busquets, Jordi García-Ojalvo, Baldomero Oliva, Francisco J Muñoz

**Affiliations:** Laboratory of Structural Bioinformatics (GRIB), Faculty of Health and Life Sciences, Universitat Pompeu Fabra, 08003 Barcelona, Spain; Laboratory of Molecular Physiology, Faculty of Health and Life Sciences, Universitat Pompeu Fabra, 08003 Barcelona, Spain; Laboratory of Molecular Physiology, Faculty of Health and Life Sciences, Universitat Pompeu Fabra, 08003 Barcelona, Spain; Laboratory of Molecular Physiology, Faculty of Health and Life Sciences, Universitat Pompeu Fabra, 08003 Barcelona, Spain; Laboratory of Molecular Physiology, Faculty of Health and Life Sciences, Universitat Pompeu Fabra, 08003 Barcelona, Spain; Laboratory of Molecular Physiology, Faculty of Health and Life Sciences, Universitat Pompeu Fabra, 08003 Barcelona, Spain; Laboratory of Molecular Physiology, Faculty of Health and Life Sciences, Universitat Pompeu Fabra, 08003 Barcelona, Spain; Barcelona Institute for Global Health (ISGlobal, Hospital Clínic-Universitat de Barcelona), 08036 Barcelona, Spain; Nanomalaria Group, Institute for Bioengineering of Catalonia (IBEC), The Barcelona Institute of Science and Technology, 08028 Barcelona, Spain; Nanoscience and Nanotechnology Institute (IN2UB), University of Barcelona, 08028 Barcelona, Spain; Laboratory of Dynamical Systems Biology, Faculty of Health and Life Sciences, Universitat Pompeu Fabra, 08003 Barcelona, Spain; Laboratory of Structural Bioinformatics (GRIB), Faculty of Health and Life Sciences, Universitat Pompeu Fabra, 08003 Barcelona, Spain; Laboratory of Molecular Physiology, Faculty of Health and Life Sciences, Universitat Pompeu Fabra, 08003 Barcelona, Spain

**Keywords:** amyloid β-peptide, insulin, insulin resistance, Alzheimer’s disease, Akt

## Abstract

Alzheimer’s disease and Type 2 diabetes are pathological processes associated to ageing. Moreover, there are evidences supporting a mechanistic link between Alzheimer’s disease and insulin resistance (one of the first hallmarks of Type 2 diabetes). Regarding Alzheimer’s disease, amyloid β-peptide aggregation into β-sheets is the main hallmark of Alzheimer’s disease. At monomeric state, amyloid β-peptide is not toxic but its function in brain, if any, is unknown. Here we show, by *in silico* study, that monomeric amyloid β-peptide 1-40 shares the tertiary structure with insulin and is thereby able to bind and activate insulin receptor. We validated this prediction experimentally by treating human neuroblastoma cells with increasing concentrations of monomeric amyloid β-peptide 1-40. Our results confirm that monomeric amyloid β-peptide 1-40 activates insulin receptor autophosphorylation, triggering downstream enzyme phosphorylations and the glucose Transporter 4 translocation to the membrane. On the other hand, neuronal insulin resistance is known to be associated to Alzheimer’s disease since early stages. We thus modelled the docking of oligomeric amyloid β-peptide 1-40 to insulin receptor. We found that oligomeric amyloid β-peptide 1-40 blocks insulin receptor, impairing its activation. It was confirmed *in vitro* by observing the lack of insulin receptor autophosphorylation, and also the impairment of insulin-induced intracellular enzyme activations and the glucose Transporter 4 translocation to the membrane. By biological system analysis, we have carried out a mathematical model recapitulating the process that turns amyloid β-peptide binding to insulin receptor from the physiological to the pathophysiological regime. Our results suggest that monomeric amyloid β-peptide 1-40 contributes to mimic insulin effects in the brain, which could be good when neurons have an extra requirement of energy beside the well-known protective effects on insulin intracellular signalling, while its accumulation and subsequent oligomerization blocks the insulin receptor producing insulin resistance and compromising neuronal metabolism and protective pathways.

## Teaser

Molina-Fernández *et al*. have demonstrated that monomeric amyloid β-peptide binds to neuronal insulin receptor mimicking insulin effect to allow glucose uptake. However, oligomeric amyloid, cause of Alzheimer’s disease, blocks insulin receptor and impairs glucose uptake. Their work proposes a physiological monomeric amyloid role and explains how oligomers contributes to neurodegeneration.

## Graphical Abstract

**Graphical abstract:**
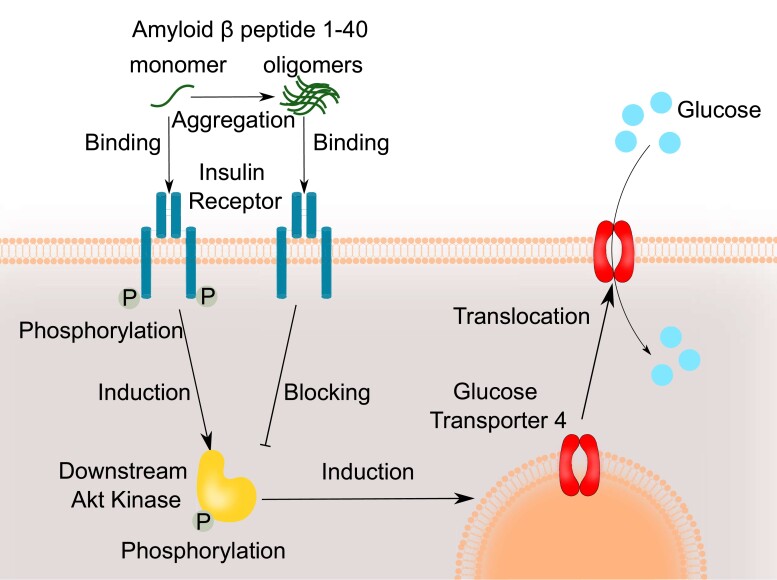

## Introduction

Alzheimer’s disease is a neurodegenerative process characterized histopathologically by extracellular senile plaques, which are insoluble aggregates of amyloid β-peptide (Aβ), and intracellular neurofibrillary tangles, composed of hyperphosphorylated protein tau.^1^ Aβ is the product of the cleavage of the amyloid precursor protein (APP), a ubiquitous protein involved in cell adhesion, synaptogenesis, axonal transport and GABA receptor regulation.^2,3^ APP is sequentially processed by the enzymes β- and γ-secretases, which release different Aβ species,^4,5^ of which Aβ_1-40_ is the most abundant.^6–8^ mAβ is not toxic, but no specific physiological function on neurons has been reported. Aβ is produced throughout life, while being physiologically degraded in the brain by different enzymes^9,10^ or cleared across the blood brain barrier into the blood to be degraded by liver.^11^ During ageing, the activity of β secretase increases, favouring Aβ production,^12^ whereas its degradation and clearance from the brain decrease.^7^ This scenario promotes the aggregation of Aβ into β sheets, forming the highly cytotoxic oligomers that can progress into large amyloid fibrils, which are packed into senile plaques.

Recent works have reported that glucose metabolism is altered long before the first symptoms of Alzheimer’s disease appear.^13,14^ Glucose uptake by neurons is mainly driven by the Glucose Transporter (GLUT) 3,^15^ but in certain brain areas such as the hippocampus (which is drastically affected by Alzheimer’s disease,^16^ it is regulated by insulin associated to the transporter GLUT4.^17^ Insulin resistance, a pathological state characterized by the impairment of insulin to induce intracellular signalling,^18^ has been reported in Alzheimer’s disease,^19^ prompting some authors to refer to Alzheimer’s disease as Type 3 diabetes.^20,21^

The insulin receptor (IR) is a tetramer formed by two dimers. Each dimer has a subunit alpha (extracellular domain) and a subunit β (transmembrane and cytosolic domain) bound covalently by disulphide bridges.^22^ A single molecule of insulin binds to the IR by interacting with the two alpha subunits, inducing the formation of the dimer that auto-phosphorylates in a tyrosine kinase domain, which activates the intracellular signalling cascade by phosphorylating the endogenous insulin receptor substrates (IRSs) of tyrosine kinase.^23^ This leads to phopshatidyl inositol 3 kinase (PI3K)/Akt phosphorylation,^24^ which triggers different downstream effects such as GLUT4 translocation to the membrane, cell metabolism, anti-apoptotic processes and gene regulation.^25^ Impaired IR signalling, on the other hand, leads to insulin resistance and neuronal damage.^26,27^ Here we have addressed the study of the Aβ binding to IR when Aβ is at the monomeric state and when it aggregates forming oligomers, by combining *in silico*, *in vitro* and systems biology approaches.

## Materials and methods

### Structures and sequences

The structure of insulin–IR was taken from Protein Data Bank (PDB), with code 6CE9,^28^ and it had been obtained by single particle Cryo-Electron Microscopy high-resolution analysis. This structure belongs to the ectodomain of the IR, between residues 28 and 758. The structures of mAβ_1-40_ (1AML)^29^ and oAβ_1-40_ (2LNQ)^30^ were taken from PDB and both had been obtained by nuclear magnetic resonance. The sequence of the IR was taken from UniProt,^31^ with code P06213 (INSR_HUMAN).

### mAβ-IR and insulin–IR supervised docking

Due to similarities between the structures of mAβ_1-40_ and insulin, we hypothesized that the binding with the ectodomain of IR dimer (PDB code 6CE9)^28^ would also be similar. We guided the docking of the structure of mAβ_1−40_ in the position of insulin, identifying hydrogen bonds and aromatic contacts that would benefit the interaction (i.e. F66, F524 and F741, these are F39 in chain A, F497 in chain B and F714 in chain P). Then, we relaxed the structure by simulated annealing, generating 100 models with the programme MODELLER^32–35^ that were further optimized with five cycles of the programme RELAX^36–39^ from the Rosetta package. The structure of the insulin–IR complex was also relaxed with the same protocol, obtaining 100 models, in order to compare the distribution of energies of both complexes (i.e. mAβ-IR and insulin–IR).

### mAβ-IR and insulin–IR unsupervised docking

We manually docked the structure of mAβ_1-40_ in the position of the insulin, forcing those near contacts benefiting the interaction, and then applied a Monte Carlo-based multi-scale docking algorithm following the Docking Protocol of the Rosetta Package.^40–45^ For the sake of comparison, the protocol was applied to both the mAβ_1-40_-IR model and the Insulin–IR native structure, generating 100 poses of each one. Finally, all poses and models were optimized with five cycles of the programme RELAX^36–39^ from Rosetta package.

### Theoretical calculation of binding energies

The binding energies of the models of mAβ_1-40_–IR and Insulin–IR in their poses, obtained by supervised and unsupervised methods, were calculated with the programmes ZRANK,^46^ InterfaceAnalyzer from the Rosetta Package^47^ and FoldX.^48^ As we used several poses, we obtained a distribution of energies for each method.

### Prediction of regions of aggregation

We hypothesized that the flexible region at the C-terminal tail of the IR ectodomain (IRct), between residues 718 and 746 of IR, has the potential to aggregate because it changes the conformation to a α-helix upon binding of insulin and dimerization. The sequence of IRct was analyzed with the programmes of prediction of ‘hot spots’ of aggregation Aggrescan,^49^ Pasta2.0^50^ and Tango.^51–53^ Results from the three services were used to select the amino acid sequence with highest probability of aggregation (Supplementary Figure 1). The sequence that was predicted to aggregate is defined as the common result of most predictions. This sequence was found between the residues D734 and R744 of P06213 (D707 and R717 in chains M and P of the structure with code 6CE9 from PDB).

### Modelling of the IR_Ct_ region embedded by oAβ_1-40_

We hypothesized that the aggregation of the IR_Ct_ region is facilitated by the oligomeric form oAβ_1-40_. We threaded the sequence predicted to aggregate into the structure of oAβ_1-40_. As the best alignment of the threading is unknown, we scanned all possible solutions by sliding the sequence predicted into the template sequence of a single chain of oAβ_1-40_ (Supplementary Figure 2). We generated five models for each pairwise alignment with the programme MODELLER^32–35^. The side chain positions were optimized with FIXBB from the Rosetta Package^54–57^. We analyzed the energies of the models with the scoring programmes ZRANK,^46^ InterfaceAnalyzer from the Rosetta Package^47^ and FoldX.^48^ We selected the final model with the best energy and compared the result with the energies of the native structure of oAβ_1-40_ to validate the viability of the complex.

### mAβ_1-40_preparation

1 mg lyophilized Aβ_1-40_ wild-type, Dutch and Scrambled (Anaspec) were solubilized in 250 µL of MilliQ water. The pH was adjusted to ≥10.5 with 1 M NaOH to avoid the isoelectric point of Aβ. 250 µL of 20 mM phosphate buffer (pH 7.4) were added to neutralize pH and samples were sonicated for 1 min in a bath-type sonicator at medium intensity (Bioruptor, Diagenode). Aliquots were prepared and dissolved to 0.4 mg/mL (92.4 µM) in serum-free Ham’s F12 GlutaMax (F12 medium; Gibco) to treat cells. Aliquots were freeze-dried and stored at −20°C until used.

### oAβ_1-40_preparation

Aβ_1-40_ was prepared as described above and incubated for 24 h at 4°C without agitation.^58^ Aliquots were prepared and dissolved to 0.4 mg/mL (92.4 µM) in serum-free F12 medium to treat cells. Aliquots were kept at −20°C until used.

### Transmission electron microscopy

mAβ_1-40_ (wild-type, Dutch and Scrambled) and oAβ_1-40_(wild-type) samples were analyzed by TEM. We used Silicon/Formvar-coated 300-mesh copper grids (Electron Microscopy Sciences), which were rendered hydrophilic by the exposition to UV light for 20 min. Samples were previously gently agitated and we put 5 μL per sample onto the grids allowing their adsorption for 2 min adsorption time. The excess was drawn off using Whatman 541 filter paper (GE Healthcare). Grids were stained with filtered 2% Uranyl Acetate (Electron Microscopy Sciences) for 1 min. Excess was drawn off and grids were air-dried. Samples were analyzed with a JEOL 1011 transmission electron microscope at an operating voltage of 80 KV.

### Transthyretin aggregation

Oligomeric transthyretin (TTR) amyloid was used as negative control for IR binding. We followed a protocol to aggregate TTR previously reported.^59^ TTR-L55P, a highly aggregative form of TTR was solubilized at a final concentration of 1 mg/mL in a solution containing 10 mM sodium phosphate, 100 mM KCl, and 1 mM EDTA (pH = 7.0). 20 µL of soluble TTR was mixed with 30 µL of the same solubilizing solution plus 50 µL of a solution containing 200 mM sodium acetate, 100 mM KCl, and 1 mM EDTA (brought to pH = 4.18 with 10 N HCl). This resulted in a TTR-L55P concentration of 0.2 mg/mL and a final pH of 4.3, the optimal pH for its aggregation. TTR was incubated for aggregation during 4 days at 37°C without stirring.

### Cell culture

Human neuroblastoma cells (SH-SY5Y cells) were grown with F12 medium supplemented with 15% foetal bovine serum (FBS; Gibco) and 1% penicillin/streptomycin (Gibco). This cell line has been previously used as model of insulin effects in neurons^60,61^ Cells were incubated at 37°C in a humidified atmosphere of 5% CO_2_. They were plated for 48 h on 6-well plates (300 000 cells/well) for western blot (WB) studies or on 24-well plates with coverslips (30 000 cells/well) for immunofluorescence analysis.

### Cellular treatments

Growth medium was removed and cells were incubated with serum-free F12 medium for 1 h. Then, cells were treated for 10 min with 100 nM insulin (Gibco) or increasing concentrations (10, 100 and 150 nM) of mAβ_1-40_ or 30 min with increasing concentrations (10, 100 and 150 nM) of oAβ_1-40_.

### Western blot analysis

Cells were lysed on ice with 50 µL of radioimmunoprecipitation assay buffer (150 mM sodium chloride, 1.0% Triton X-100, 0.5% sodium deoxycholate, 0.1% sodium dodecyl sulphate , 50 mM Tris, pH 8.0, supplemented with 1 mM NEM, 0.1 mM PMSF, 2 mM TCEP, 1 mM sodium ortovanadate, phosphatase and protease inhibitors). Samples were lysed using a shaker for 30 min at 4°C and centrifuged at 10 000 g at 4°C for 10 min. 20 µL per sample were loaded into 4–12% Bis-Tris NuPAGE (Invitrogen). Afterwards, proteins were transferred onto 0.2 µm pore nitrocellulose membranes. Membranes were blocked for 1h at room temperature (RT) with Tween 20-Tris buffer solution (TTBS: 100 mM Tris-HCl, 150 mM NaCl, pH 7.5) plus 5% bovine serum albumin (BSA, phosphorylated fractions) or 5% skimmed milk (total fractions). Then, membranes were incubated overnight (o.n.) at 4°C with 1:500 rabbit anti-phospho(p)-IR (Tyr1361) antibody (Ab; Abcam), 1:500 mouse anti-IR Ab (Cell Signalling), 1:500 mouse anti-p-Akt (Thr308)^62^ Ab (Cell Signaling), 1:1000 rabbit anti-Akt Ab (Cell Signaling), 1:2000 mouse anti-GAPDH Ab (Abcam) horseradish peroxidase-conjugated donkey anti-rabbit and anti-mouse Abs (GE Healthcare) were used as secondary Abs at 1:2000 for 1 h at RT. Primary Abs were diluted either in 5% skimmed milk-TTBS (anti-IR, anti-Akt and anti-GAPDH) or in 5% BSA in TTBS (anti-p-IR and anti-p-Akt). Secondary Abs were diluted in TTBS. Bands were visualized with Super Signal (Pierce) and analysed with the Quantity One system in a BioRad Universal Hood II. Data were normalized by the corresponding GAPDH bands.

### Immunofluorescence studies by confocal microscopy

Human neuroblastoma cells (3 × 10^4^ cells/well) were seeded on coverslips in 24-well plates. After 3 days, the growth medium was removed. Cells were treated with 100 nM mAβ_1-40_ or oAβ_1-40_ or/and 100 nM insulin in F12 medium without FBS during 10 min. Then, cells were fixed with 4% paraformaldehyde (PFA). Regarding oligomers, cells were treated for 30 min with 100 nM oAβ_1-40_ to allow the binding of the oligomers and therefore with 100 nM insulin for 10 min in F12 medium without FBS. Cells were also fixed with 4% PFA. Fixed cells were permeabilized with 0.1% Triton X-100 and blocked with 3% BSA phosphate-buffered saline. Coverslips were incubated o.n. at 4°C with 1:100 mouse anti-p-Akt Ab (Thr 308). After three washes, coverslips were incubated with 1: 2000 Alexa Fluor 647 goat anti-mouse Ab and 1:1000 DAPI for 1h at RT. Coverslips were mounted with Fluoromount. Digital images were taken with a Leica TCS SP8 confocal microscope and analyzed with Leica confocal software.

In order to study the GLUT4 translocation to the membrane, human neuroblastoma cells were treated as described above and the immunofluorescence protocol was repeated without permeabilization. This procedure was performed using 1:100 rabbit anti-GLUT4 Ab (NBP1-49533; Novus Biologicals). Nuclei were stained with DAPI. Images were obtained by confocal microscopy using an SP8 and the maximum intensity field in a stack was chosen for depiction and measurement.

### IR binding by proximity ligation assay

Human neuroblastoma cells were treated with 1 µM mAβ_1-40_ and 1 µM oAβ_1-40_and fixed using 4% PFA. Cells were blocked according to manufacturer's instructions (DUO92101; Sigma), and incubated with 1:100 mouse 6e10 anti-APP (SIG-39340; Covance) and 1:100 rabbit anti-IR-α (NBP2-67726; Novus Biologicals) Abs. Coverslips were mounted on the slide using proximity ligand assay (PLA)’s DUOLINK *in situ* mounting medium with DAPI. Images of Aβ and IR interaction were obtained by confocal microscopy and the maximum intensity field in a stack was chosen for depiction and measurement.

### Glucose uptake assay

Human neuroblastoma cells were seeded at 20 000 cells/well in a 96 well plate. They were treated with 10, 100 and 150 nM of mAβ_1-40_or 100 nM of insulin for 10 min or pre-treated with 150 nM oAβ_1-40_ for 30 min and with/without 10 min of 100 nM of insulin. Glucose uptake was assayed following the manufactures instruction with the kit Glucose Uptake-Glo Assay (Promega). Luminescence was measured using VICTOR Nivo Multimade Plate Reader (PerkinElmer). Data are expressed as percentages regarding the untreated controls.

### Statistical analysis

Data are expressed as the mean ± SEM of the values from the number of experiments as indicated in the corresponding figures. One-way analysis of variance (ANOVA) plus Student–Newman–Keuls as *post hoc* test was used for statistical analyses setting the significant level at *P* < 0.05. The statistical analysis was performed with the open software R Licenses (GNU Affero General Public License v3).

### Data availability

The authors confirm that the data supporting the findings of this study are available from the corresponding author upon reasonable request.

## Results

### *In silico* analysis of mAβ_1-40_ binding to the extracellular IR domain as analogue of insulin

**Figure 1:**
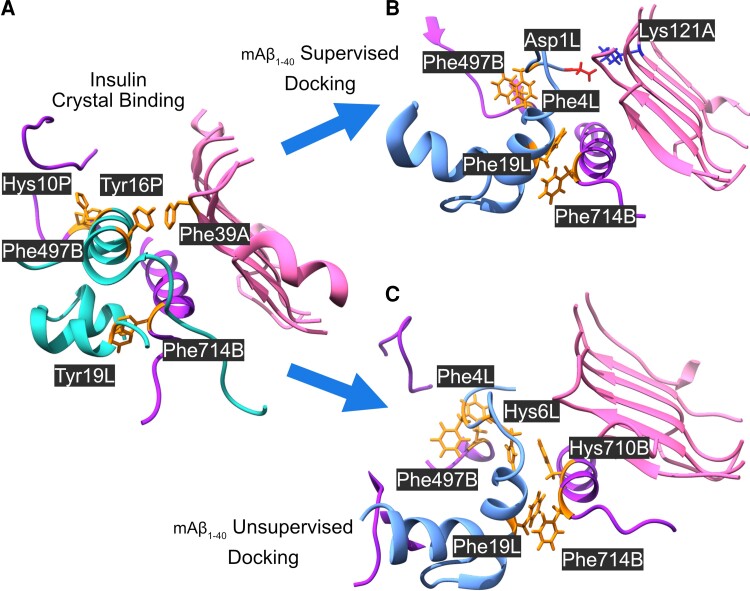
**Interactions between IR and mAβ_1-40_ and insulin.** (**A**) Ribbon plot of the relaxed structure of insulin in complex with IR, obtained with the EM structure, code 6CE9 in PDB (chains A in pink and B in purple). (**B**) IR-mAβ_1-40_binding obtained with supervised docking. (**C**) IR-mAβ_1-40_ binding obtained with unsupervised docking. Main interacting side-chains are highlighted.

**Figure 2:**
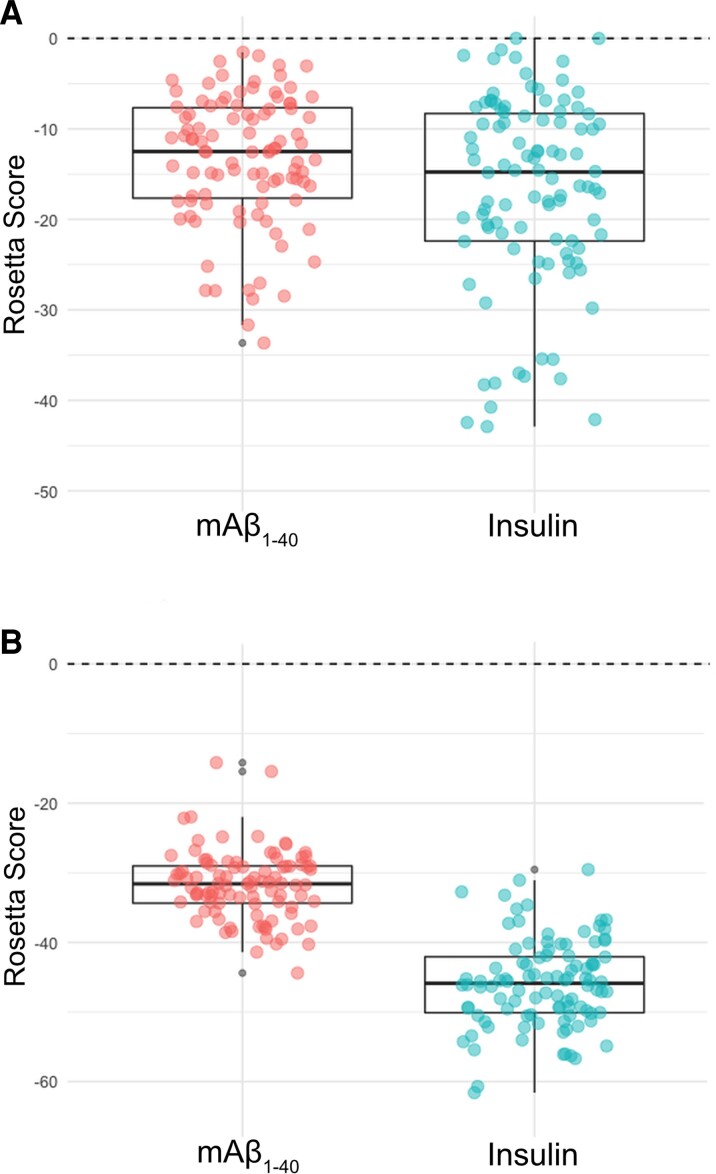
**mAβ_1-40_binds to IR with a similar energy than insulin *in silico*.** Distribution of binding energies calculated with Rosetta package (programme InterfaceAnalyzer) of Aβ soluble and insulin. Structures are derived from several relaxed models of the interaction obtained with (**A**) unsupervised or (**B**) supervised docking. *n* = 100 per each model. Non-significant differences were obtained by one-way ANOVA plus Student–Newman–Keuls as *post hoc* test.

The α subunit of IR has three regions that recognize and bind insulin. The first region is close to the N-terminus at the amino acids 20–120.^63^ The second region, and the most physiologically relevant one, is a region rich in cysteines where disulphide bridges are formed.^64^ The third region is close to the C-terminus around amino acid 390.^65^ We have performed an *in silico* comparison of the binding of insulin and soluble mAβ_1-40_to the crystallographic structure of the IRct, by supervised and unsupervised docking with Rosetta. Our model suggests that mAβ_1-40_ and insulin bind in a similar conformation to the IR tetramer according to both docking approaches (Fig. 1). We model the binding of insulin and mAβ_1-40_using the same protocols, to compare the distributions of binding energies calculated with Rosetta. Fig. 2 shows that binding occurs with sufficient affinity (ΔG < 0) in the two cases. According to the *in silico* comparison, both insulin and mAβ_1-40_ would ensure the dimerization of IR. This implies that mAβ_1-40_ could act as a functional analogue of insulin.

### *In vitro* analysis of the cellular effects of mAβ_1-40_on IR

**Figure 3:**
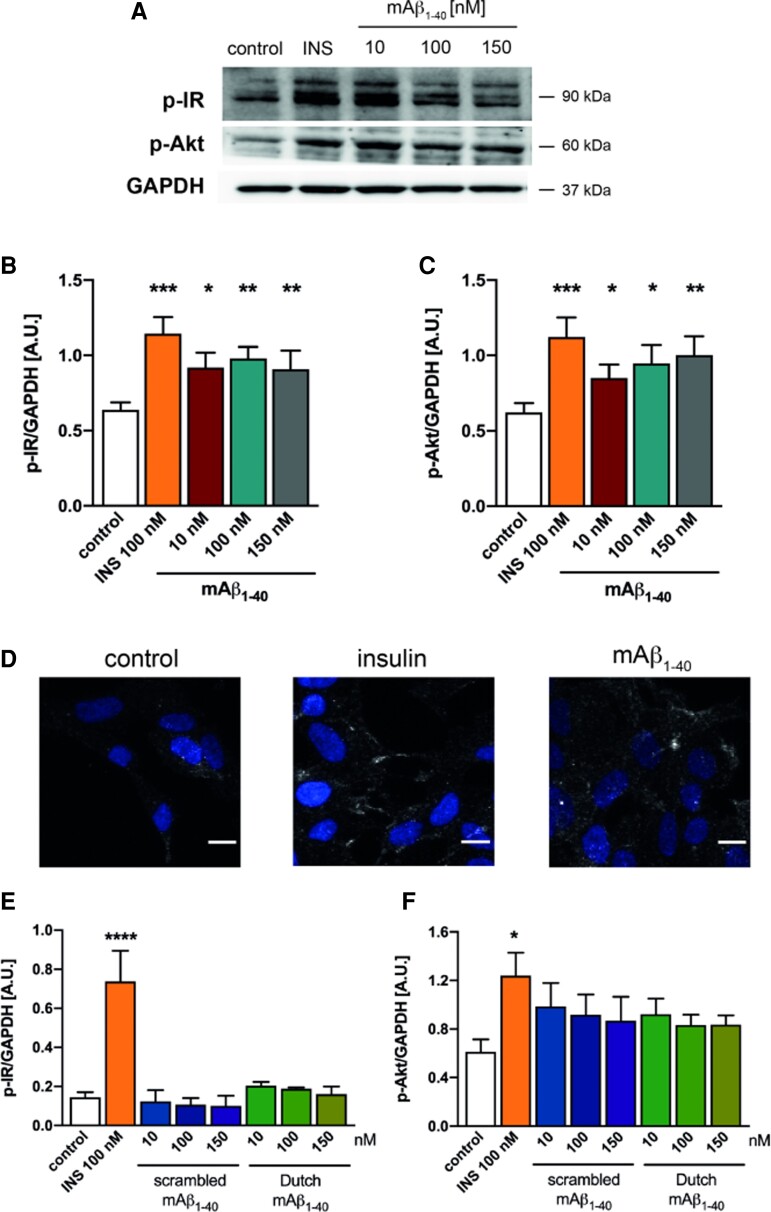
**mAβ_1-40_ mimics the insulin effect on IR**. (**A**) mAβ_1-40_binds to IR inducing its autophosphorylation. Human neuroblastoma cells were treated with increasing concentrations of mAβ_1-40_ (10, 100 and 150 nM) or physiological concentrations of insulin (100 nM) for 10 min. The upper panel shows a representative WB carried out with anti-p-IR, anti-p-Thr308-Akt and GAPDH Ab. Quantifications of p-IR and p-Akt WB bands normalized by GAPDH are shown in (**B**) and (**C**), respectively. Data are the mean ± SEM of eight independent experiments. * *P* = 0.05, ** *P* < 0.01, *** *P* < 0.001 regarding control untreated cells by one-way ANOVA plus student–Newman–Keuls as *post hoc* test. (**D**) mAβ_1-40_ induces Akt phosphorylation at Thr308 studied by immunofluorescence. Cells were treated with 100 nM mAβ_1-40_ or 100 nM insulin for 10 min. Cells were labelled with an anti-*p*-Akt Ab. Nuclei are stained with DAPI. Images were taken from a representative experiment. Bars represent 10 nm. (**E, F**) mAβ_1-40_ Dutch and Scrambled do no induce IR (**E**) or Akt (**F**) phosphorylation. Cells were treated as explained in (**A**) and assayed by WB (Supplementary Figure 6). Quantifications of p-IR and p-Thr308-Akt WB bands normalized by GAPDH are shown in (E) and (F), respectively. Data are the mean ± SEM of three independent experiments. *P < 0.05, ****P < 0.0001 regarding control untreated cells by one-way ANOVA plus Student–Newman–Keuls as *post hoc* test.

The cytosolic region of the β subunit of IR contains 13 amino acids organized in three clusters (juxtamembranous, tri-tyrosine and C-terminus) that can be phosphorylated by the tyrosine–kinase activity when insulin is bound.^66^ To verify the *in silico* prediction made above according to which mAβ_1-40_ can also bind to IR, we treated human neuroblastoma cells with 10, 100 and 150 nM mAβ_1-40_ concentrations that can be reached in human brain parenchyma.^67^ In agreement with that prediction, we have found that mAβ_1-40_ at low nanomolar ranges is able to induce significantly the IR autophosphorylation (Fig. 3A and B) at a level comparable with 100 nM insulin. These treatments do not affect to the total levels of IR (Supplementary Figure 3). The mAβ_1-40_ samples were analyzed by transmission electron microscopy (TEM) to discard the presence of oligomers or aggregated species of Aβ (Supplementary Figure 4A). We also tested these samples to demonstrate their lack of neurotoxicity by cell survival assay (Supplementary Figure 5).

We next asked whether the downstream effects of mAβ_1-40_ on IR signalling mimic those of insulin. IR autophosphorylation activates IRS-1 and IRS-2, thereby triggering different signalling pathways. The phosphorylation of the enzyme Akt is the most relevant since it induces the translocation of GLUT4 to the membranes. We thus studied the effect of mAβ_1-40_on Akt phosphorylation, obtaining that Akt is phosphorylated significantly with increasing mAβ_1-40_ concentrations (Fig. 3A, C and D), similar to the results obtained with 100 nM insulin and without affecting to the total levels of Akt (Supplementary Figure 3).

**Figure 4:**
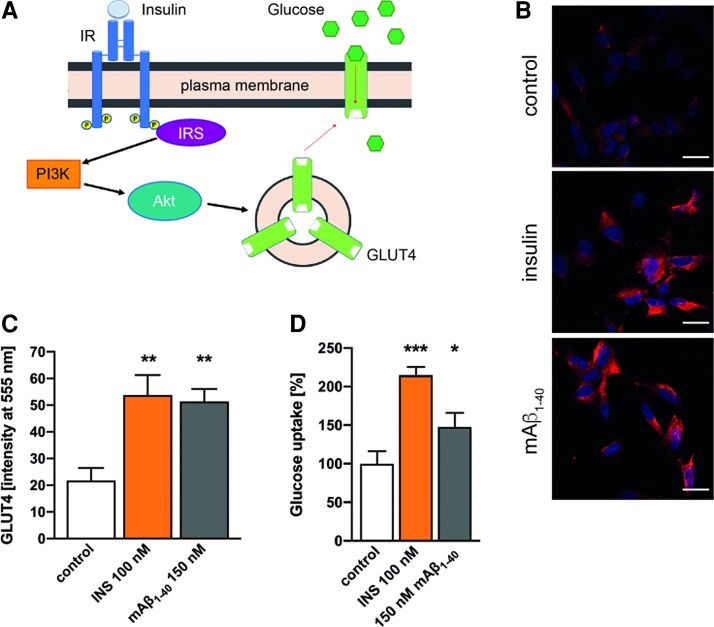
**mAβ_1-40_ induces GLUT4 membrane translocation.** (**A**) Scheme of the intracellular effects of IR activation. Insulin binds the IR inducing its dimerization and activating the receptor to cause its autophosphorylation. This produces the binding and phosphorylation of the signalling adapter protein IRS. IRS activates several downstream pathways, but here we focus on PI3K/Akt system. The activation of PI3K leads to the phosphorylation of Akt, which produces the GLUT4 translocation to the membrane, allowing glucose uptake by cells. (**B, C**) mAβ_1-40_induces GLUT4 translocation. Cells were treated for 10 min with 100 nM insulin or 150 nM mAβ_1-40_, afterwards cells were fixed. Extracellular expressed GLUT4 was labelled with an anti-GLUT4 Ab and nuclei were stained with DAPI. (**B**) Representative images of cells used in (**B**). Bars represent 20 nm. (**C**) GLUT4 fluorescence intensity at 555 nm was quantified. Data are the mean ± SEM of 4 independent experiments. ** *P* < 0.01 compared with untreated controls by one-way ANOVA plus Student–Newman–Keuls as *post hoc* test. (**D**) Cells were treated as in (**B, C**). Glucose uptake was measured and expressed regarding untreated controls. Data are the mean ± SEM of five independent experiments. ***P < 0.01, *P < 0.05 compared with controls by one-way ANOVA plus Student–Newman–Keuls as *post hoc* test.

Furthermore, we have used two types of mAβ_1-40_with different sequences to that of the wild-type to test the specificity of the mAβ_1-40_wild-type on IR activation. They are the synthetic mAβ_1-40_Scrambled and the mutated mAβ_1-40_type Dutch, which has a substitution of a Glu by Gln at position 21 yielding to a very early onset of Alzheimer’s disease^68^ (Fig. 3E and F; Supplementary Figure 6). As expected, these Aβ_1-40_do not induce IR nor Akt phosphorylation (Fig. 3E and F). These treatments do not affect to the total expression of IR nor Akt (Supplementary Figure 6). The mAβ_1-40_ Dutch and Scrambled samples were also analyzed by TEM to discard the presence of amyloid structures (Suplementary Fig. 4C and D) that would interfere with the binding to IR.

The main physiological effect of insulin is the final translocation of the GLUT4 to the membrane allowing the glucose entrance upon IR and AKT phosphorylation (Fig. 4A). Therefore, we have studied whether mAβ_1-40_is also able to induce the translocation of GLUT4 to the membrane (Fig. 4B and C). We have found that mAβ_1-40_induces the GLUT4 membrane expression significantly (*P* < 0.01). These findings suggest that mAβ_1-40_ mimics the classical intracellular signalling of insulin. We tested if insulin, the strongest regulatory stimulus of GLUT4, has any effect on total GLUT4 expression levels and it was discarded (Supplementary Figure 7). Finally, we studied the functional role of the effect of mAβ_1-40_ on GLUT4 by measuring the glucose uptake (Fig. 4D). As expected, mAβ_1-40_promotes the glucose uptake (*P* < 0.05).

### *In silico* analysis of oAβ_1-40_ binding to the extracellular IR domain as antagonist of insulin

**Figure 5:**
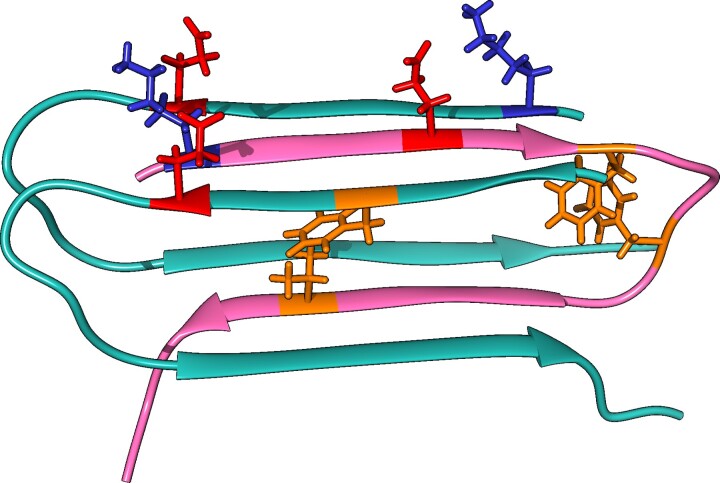
**Model of the aggregate-predicted sequence of IR_Ct_, embedded by two chains of Aβ_1-40_forming an oligomer conformation.** Ribbon plot of the IR_ct_ model of the sequence predicted to aggregate. The sequence aggregates within a subunit of the oAβ_1-40_ structure. Relevant residues stabilizing the fibre are highlighted (positively charged in blue and negatively charged in red, aromatic residues shown in orange).

Having shown that mAβ_1-40_mimics insulin binding to the IR with similar downstream effects, we next turned to the oligomeric form of the peptide, more commonly associated with the early stages of Alzheimer’s disease. To understand the role of oAβ in insulin signalling, we have analyzed the sequence of the C-terminal tail of IRct (residues 718–746 of IR). IR_Ct_ is a highly flexible domain with no regular secondary structure, which changes its conformation into an α−helix upon binding of insulin and dimerization. We hypothesize that such a flexible region could aggregate when not in the conformation of α−helix, based on the prediction of algorithms such as Tango,^51–53^ Pasta2.0,^50^ and Aggrescan^49^ (see Supplemenary Table 1 and details in the STAR Methods section). To test the potential aggregation involving IR_Ct_, we modelled the structure of the sequence of the fragment with highest probability to aggregate. The sequence was threaded on the β-conformation of oAβ_1-40_ (template structure 2LNQ)^30^ until fitting the location of the Aβ_1−40_ side-chains with the lowest energy on the interface of the oligomers (see details in the STAR Methods section). Fig. 5 shows the orientation of the side-chains of aromatic and hydrophobic residues towards the core, while K16 and E22 form hydrogen bonds with the same residues of the other subunits. The interaction of the aggregation segment of IR_Ct_, embedded by two chains of Aβ, showed a large negative binding energy (Supplementary Figure 2), suggesting that IR_Ct_might act as a seed of nucleation of Aβ_1-40_when its concentration increases. In turn, the formation of the oligomeric form of Aβ_1−40_ complexed with the IR_Ct_ fragment would strongly compete with the formation of the α−helix and the dimerization of IR that would otherwise be induced by insulin binding. This results in oAβ_1-40_ acting as an antagonist of insulin in the binding to IR, and thereby impairing downstream insulin signalling.

### Kinetic model of the competition between insulin and Aβ

**Figure 6:**
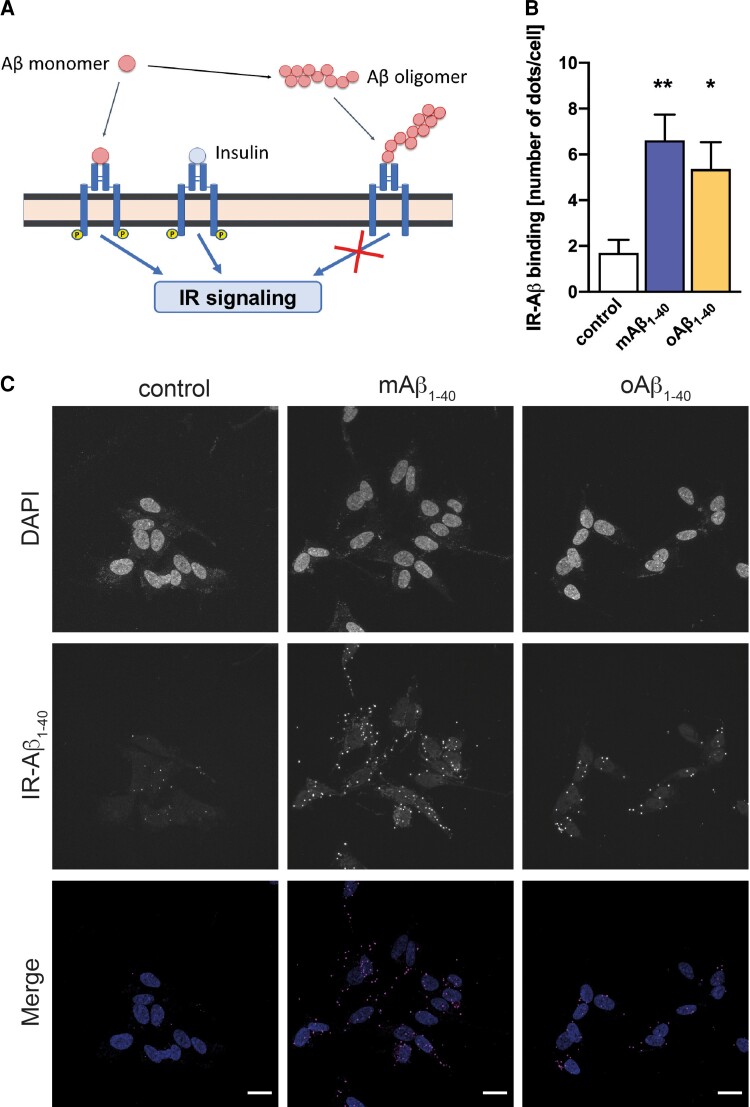
**Aβ_1-40_ binds to IR.** (**A**) Modelling the competition dynamics for insulin signalling. Scheme showing the main components of the system and their interactions considering the physiological activation of IR by mAβ_1-40_and insulin and the antagonist role of oAβ_1-40_ in insulin signalling. (**B**) Human neuroblastoma cells were treated with 1 µM mAβ_1-40_or 1 µM oAβ_1-40_ for 10 and 30 min, respectively. Binding assay (PLA) was carried out as indicated in the Materials and Method section. Data are the mean ± SEM of the quantification of the dots obtained by the binding assay in six independent experiments demonstrating Aβ-IR interaction. * *P* < 0.05; ** *P* < 0.01 by one-way ANOVA plus Student–Newman–Keuls as *post hoc* test. (**C**) Representative images of mAβ_1-40_or oAβ_1-40_ and IR interaction obtained by binding assay. Upper panels show nuclei using DAPI, mid panels show dots of PLA interactions, and lower panels show merge were PLA interactions are depicted. Bars represent 10 nm.

Our experimental observations indicate that mAβ_1-40_ can mimic insulin, both in its ability to bind the IR and in its capacity to activate the corresponding signalling cascade. We propose that oAβ_1-40_, in turn, can also bind IR but does not trigger signalling (Fig. 6A). Thus, the three species (insulin, mAβ and oAβ_1-40_) compete for the same ‘resource’ (IR), with different effects. Additionally, mAβ_1-40_can become part of oAβ_1-40_ by aggregation with existing oligomers. These interactions are summarized by the set of biochemical reactions shown in Supplementary Figure 8A. The pathophysiological relevance of this hypothesis has been tested by the performance of binding assays (Fig. 6B and C). We have demonstrated that both mAβ_1-40_and oAβ_1-40_ bind to IR significantly (*P* < 0.01 and *P* < 0.05, respectively).

We can describe the dynamics of all the biochemical species that participate in the reactions above by using the law of mass action. Further assuming that the complexes IR:Ins, IR:mAβ and IR:oAβ are in quasi-steady state, we can obtain a reduced set of ordinary differential equations for the concentrations of the free species: insulin (I), mAβ (M), oAβ (Α) and the IR itself (R) as shown in Supplementary Figure 8B. We have obtained two solutions (Supplementary Figure 8B, equations S8 and S9), but solution (S9) is only biologically meaningful when A_st_ and M_st_ are non-negative (obviously the same applies to R_st_ and I_st_ in expressions). Additionally, a linear stability analysis shows that the solutions are stable only when they are positive, as shown in Supplementary Figure 9. This implies that only one equilibrium is stable for any value of the control parameter taken to be the monomer production rate α_m_ in Supplementary Figure 9. For a critical value of that parameter (vertical dashed line in Supplementary Figure 9), the two equilibria collide and exchange their stability. This is the hallmark of a transcritical bifurcation.

**Figure 7:**
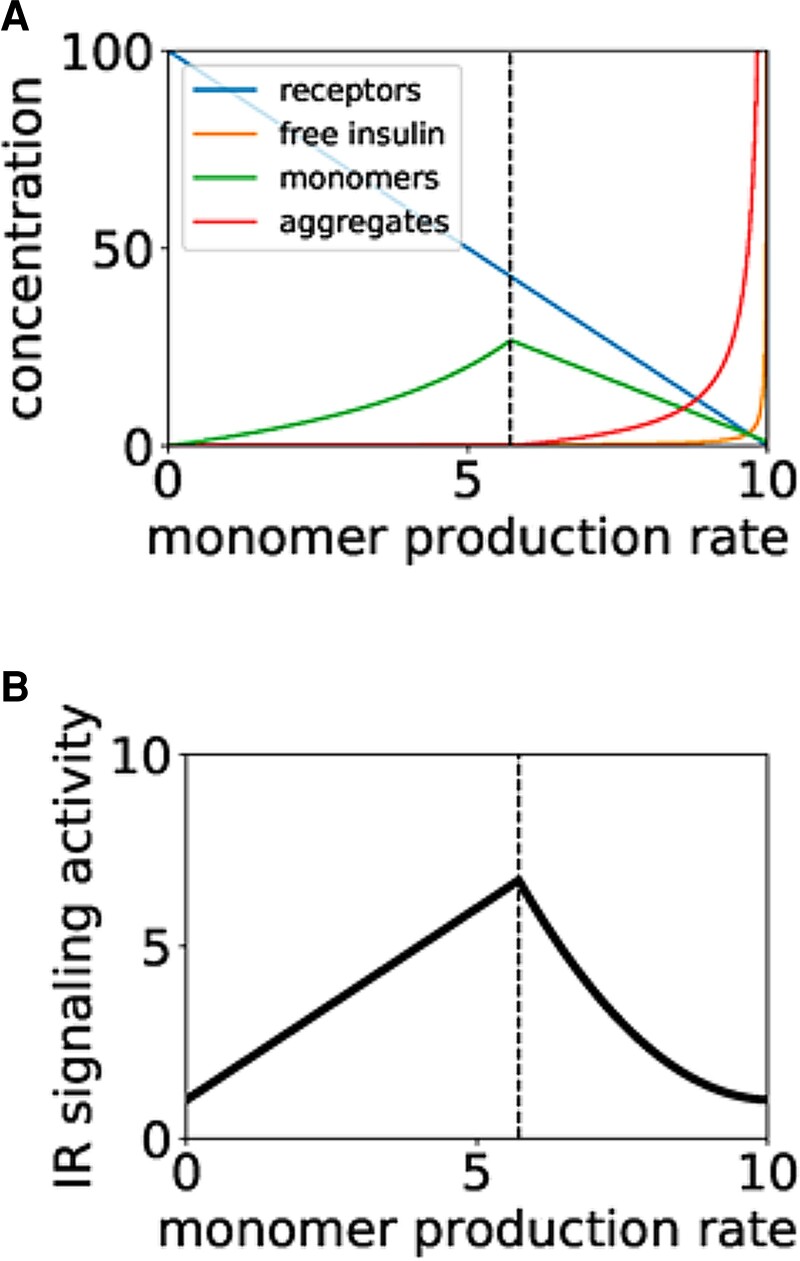
**Steady-state of our model as a function of the monomer production rate, α_m_.** (**A**) Steady-state concentrations of the different species. (**B**) Activity of the signalling pathway for increasing α_m_. The vertical dashed line in both plots represents the location of the transcritical bifurcation described in Supplementary Figure 9. Parameters used in A are those given in Supplementary Table 2. All parameters and variables are in arbitrary unit.

The behaviour of all model variables across the bifurcation is shown in Fig. 7A, which represents the stable equilibrium as a function of α_m_. As can be seen in the plot, at the left of the transcritical bifurcation point (vertical dashed line) the oAβ_1-40_ (aggregate, red line; Fig. 7B) is zero, becoming non-zero at the bifurcation point. At that moment, the mAβ_1-40_(green line) starts to decrease. The receptor concentration (blue line) decreases monotonically as α_m_ grows, due to its increased loss by monomer binding. The free insulin (yellow line) also increases, slowly at first and quickly when α_m_ (and the aggregates) become high enough, due to increased competition for binding to the receptor. Finally, Fig. 7B shows the effect of the bifurcation on downstream IR signalling: the activity of the IR signalling pathway initially increases, as long as the aggregates are absent, due to the effect of both insulin and mAβ_1-40_binding to IR. After the bifurcation, in contrast, signalling activity starts to decrease due to futile binding of the receptor to the oligomeric form of Aβ.

Finally, we studied how the critical value of the monomer production rate, α_m_, at which the transcritical bifurcation happens (and thus insulin signal peaks) depends on all the biochemical processes (Fig. 6A). The results, plotted in Supplementary Figure 10, show that IR signalling is more robust (i.e. the bifurcation happens for a higher monomer production rate) when (i) the receptor production rate increases, (ii) the insulin production rate decreases,^67,69^ (iii) the intrinsic endocytic rate of the receptor decreases, (iv) the binding rate of both monomers and aggregates to the IR increases, (v) the aggregation rate decreases, and (vi) the deaggregation rate increases. Low insulin production, for instance, alleviates competition for the receptor, which can titrate more monomer molecules. In this situation, a higher production rate of monomers is needed to trigger aggregation. The critical monomer production rate also grows with increasing binding rate of the aggregates to the receptor. We interpret this result by considering that when the receptor titrates aggregate molecules more easily, more monomer production is needed to cause the existence of free aggregates. Interestingly, for high enough aggregate binding rate the model produces an oscillatory behaviour, as shown in Supplementary Figure 1^1^. On the other hand, the model predicts no dependence of the bifurcation point on the binding rate of the insulin to the IR.

### *In vitro* impairment of insulin signalling by oAβ_1-40_

**Figure 8:**
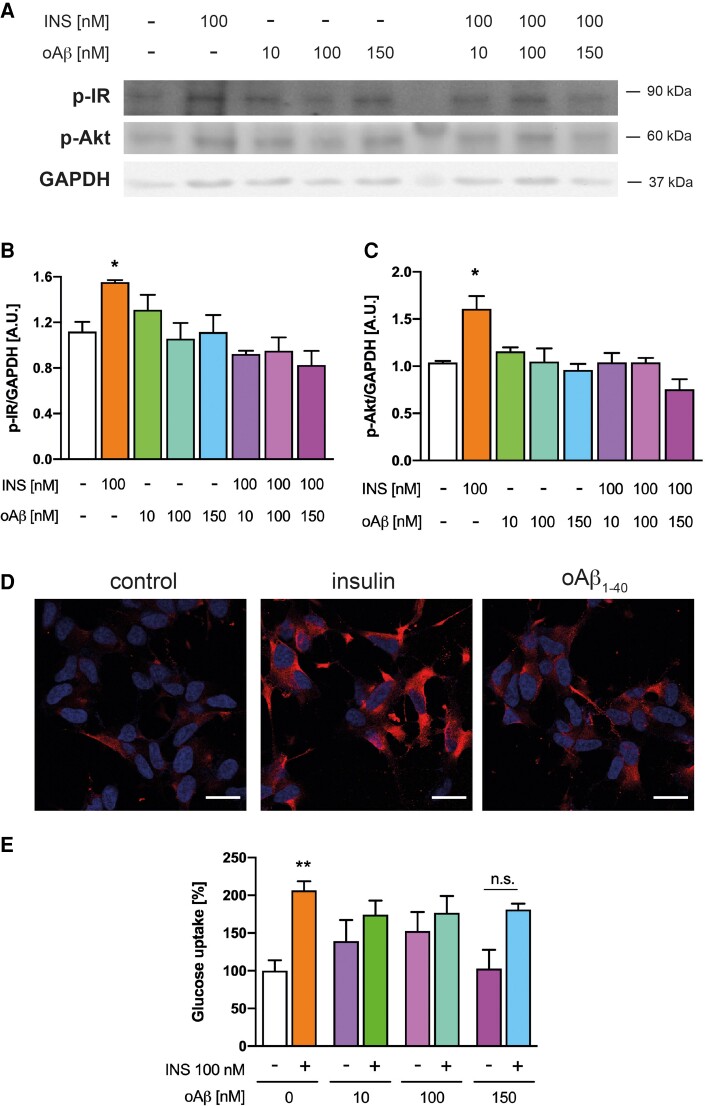
**oAβ_1-40_blocks IR activation.** (**A**) oAβ_1-40_binds to IR impairing its autophosphorylation. Human neuroblastoma cells were pre-treated with increasing concentrations of oAβ_1-40_for 30 min and then treated in the presence/absence of insulin (100 nM) for 10 min. The upper panel shows a representative WB with anti-p-IR, anti-p-Thr308-Akt and anti-GAPDH Abs. (**B**) Quantification of the p-IR bands and normalized by GAPDH obtained by WB as described in (**A**). Data are the mean ± SEM of 4–5 independent experiments. *P < 0.05 compared with untreated control cells by one-way ANOVA plus Student–Newman–Keuls as *post hoc* test. (**C**) Quantification of the p-Thr308-Akt bands and normalized by GAPDH obtained by WB as described in (**A**). Data are the mean ± SEM of 4–5 independent experiments. *P < 0.05 regarding untreated control cells by one-way ANOVA plus Student–Newman–Keuls as *post hoc* test. (**D**) oAβ_1-40_do not induce the translocation of GLUT4. Representative images of cells treated with 100 nM insulin and 150 nM oAβ_1-40_ for 10 and 30 min, respectively. Extracellular GLUT4 was labelled with an anti-GLUT4 Ab and nuclei are stained with DAPI. Bars represent 20 nm. (**E**) oAβ_1-40_ does not induce glucose uptake. Cells were treated as in (**A**) and glucose uptake was measured as indicated in the M&M section and expressed regarding untreated controls. Data are the mean ± SEM from five independent experiments. ** *P* < 0.01, n.s., non-significant compared with untreated cells by one-way ANOVA plus Student–Newman–Keuls as *post hoc* test.

To test the hypothesis that oAβ_1-40_ impairs insulin signalling, we induced the oligomerization of Aβ_1-40_*in vitro* as previously described.^58^ We next treated cells with oAβ_1-40_, confirming that oAβ_1-40_ by itself does not induce IR phosphorylation (Fig. 8A and B). In the presence of insulin, on the other hand, oAβ_1-40_ acted as a blocker of IR signalling, since increasing concentrations of oAβ_1-40_ impaired IR activation proportionally to its concentration. To verify the downstream implications of this inhibition, we studied the effect of oAβ_1-40_ on Akt activation. As expected, no Akt activation was induced (Fig. 8C) nor GLUT4 translocation to the membrane (Fig. 8D) neither glucose uptake (Fig. 8E). Consistently, we observed that oAβ_1-40_ impairs the effect of insulin on the physiological IR intracellular signalling. We used TTR oligomers as controls to study the specificity of oAβ_1-40_ to impair Akt phosphorylation by insulin (Supplementary Figure 1^2^). As expected, TTR did not prevent Akt phosphorylation when insulin was added to the cells. Finally, the oAβ_1-40_ samples were analyzed by TEM to demonstrate the presence of oligomers (Supplementary Figure 4B).

## Discussion

Alzheimer’s disease patients show an impairment in neuronal glucose uptake,^13,14^ which results in energy depletion. This has been related to insulin resistance, which is mainly caused by the functional uncoupling of the IR with its intracellular signalling pathway. Insulin resistance affects energy production, but also neuronal survival, gene expression and neuronal plasticity.^70^ The latter two effects are tightly linked to memory formation and learning, processes that take place in the hippocampus, the brain centre most severely damaged in Alzheimer’s disease due to the high Aβ load.^71,72^

Aβ is neurotoxic when it aggregates into oligomers and fibrils in the brain.^73–75^ Although Aβ is produced in its monomeric state in the brain at a constant rate since childhood,^76,77^ its physiological function is not known. Our *in silico* analysis and experimental data show that after treatment with mAβ_1-40_, IR and Akt phosphorylation are increased. This effect is physiologically relevant for neurons, since it suggests that mAβ_1-40_could be working as an agonist of the IR, helping insulin to regulate glucose uptake. Insulin is a hormone whose entrance in the brain across the blood brain barrier is mediated by saturable mechanisms.^78,79^ We conjecture that Aβ could help glucose intake in neurons during periods of high neuronal activity and deficient insulin delivery to the brain. A physiological convergence between the functions of amyloid and insulin would also be expected from the fact that one of the two main enzymes that degrades Aβ in brain is the Insulin Degrading Enzyme (IDE).^10^ Moreover, Akt is the main downstream effector of IRS activation. Since Akt is involved in a wide range of regulatory processes, including the activation of anti-apoptotic proteins, the inhibition of pro-apoptotic proteins, cell cycle progression, and the trigger of metabolic processes,^80^ the observed Akt activation would suggest a protective mechanism for mAβ_1-40_.

On the other hand, Alzheimer’s disease has been proposed as a metabolic disease due to insulin resistance in the brain.^20,21^ Previous works have proposed the IR as a target for aggregated Aβ,^81,82^ reflected in a high IR endocytic rate when cells are treated with fibrillar Aβ for long times. We have found, by *in silico* modelling, kinetic dynamic analysis and *in vitro* data, that oAβ_1-40_ works as a blocker of IR, impairing the activity of the insulin in neurons. Specifically, IR is not phosphorylated when oAβ_1-40_ is present. Consequently, Akt activation is also impaired. This would have three major consequences in neurons: (i) a lower entrance of glucose, (ii) lack of the protective effects of Akt, making these neurons more sensitive to the damage caused by oAβ_1-40_ in other membrane components and (iii) endocytosis of the inefficient IR, contributing to insulin resistance by receptor down-regulation.

Summarizing, we propose that mAβ_1-40_, the Aβ species mostly produced along the life by neurons, has a physiological role mimicking the effect of insulin in the brain areas that requires exceptional glucose entrance as hippocampus and cortex. On the other hand, when Aβ in excess aggregates to form oAβ, it contributes to generate insulin resistance in the brain. This might provide a molecular correlation to the observed co-morbidity between Alzheimer’s disease and diabetes as individuals age.

## Supplementary Material

fcac243_Supplementary_Data

## Abbreviations

Aß =: Amyloid ß-peptide
Ab =: antibody
APP =: amyloid precursor protein
BSA =: bovine serum albumin
FBS =: foetal bovine serum
GLUT =: glucose transporter
IR =: insulin receptor
IRS =: insulin receptor substrate
p- =: phospho-
o.n. =: overnight
PDB =: Protein Data Bank
PFA =: paraformaldehyde
PI3K =: phopshatidyl inositol 3 kinase
PLA =: proximity ligand assay
RT =: room temperature
TEM =: transmission electron microscopy
TTR =: transthyretin
T2D =: Type 2 diabetes
WB =: western blot
