# Supplementary material for: Differential regulation of insulin signalling by monomeric and oligomeric amyloid beta-peptide
Source: Brain Commun. 2022 Sep 24;4(5):fcac243. doi: 10.1093/braincomms/fcac243 (PMC9576151; doi:10.1093/braincomms/fcac243)
Supplement: fcac243_Supplementary_Data [file fcac243_supplementary_data.pdf]

SUPPLEMENTARY MATERIAL

Supplementary Table 1 Prediction of "hot spot" fragments of aggregation in the IR_{ct} sequence

Resource	Method	Hit	Source
**Webserver**			
Pasta2.0	Machine Learning plus Statistical Energy function	LKELEESSFRKT FE**DYLHNVVFV** PRKTS	Department of Biomedical Sciences, University of Padova
Aggrescan	Libraries of fragments obtained by in vivo aggregation experiments	LKELEESSFRKT FEDYL**HNVVFV** PRKTS	Universitat Autònoma de Barcelona (UAB)
Tango	Boltzmann distribution	LKELEESSFRKT FEDYLH**NVVFV** **PR**KTS	Centre of Genomic Regulation (CRG)

Web servers used for the prediction of the aggregation region of Irct. Columns indicate the web server used, the method of prediction, the predicted fragment in bold letter and source of the web server.

Supplementary Table 2 Parameter values used in the kinetic modelling

Parameter	Description	Value (Arb. Units)
α_r	Receptor production rate	11
α_m	Monomer production rate	8.5
α_i	Insulin production rate	1
k_i	Insulin binding rate	0.1
k_m	Monomer binding rate	0.005
k_a	Aggregate binding rate	0.06
k_b	Aggregation rate	0.1
k_u	Deaggregation rate	0.1
δ_r	Intrinsic receptor endocytosis rate	0.1

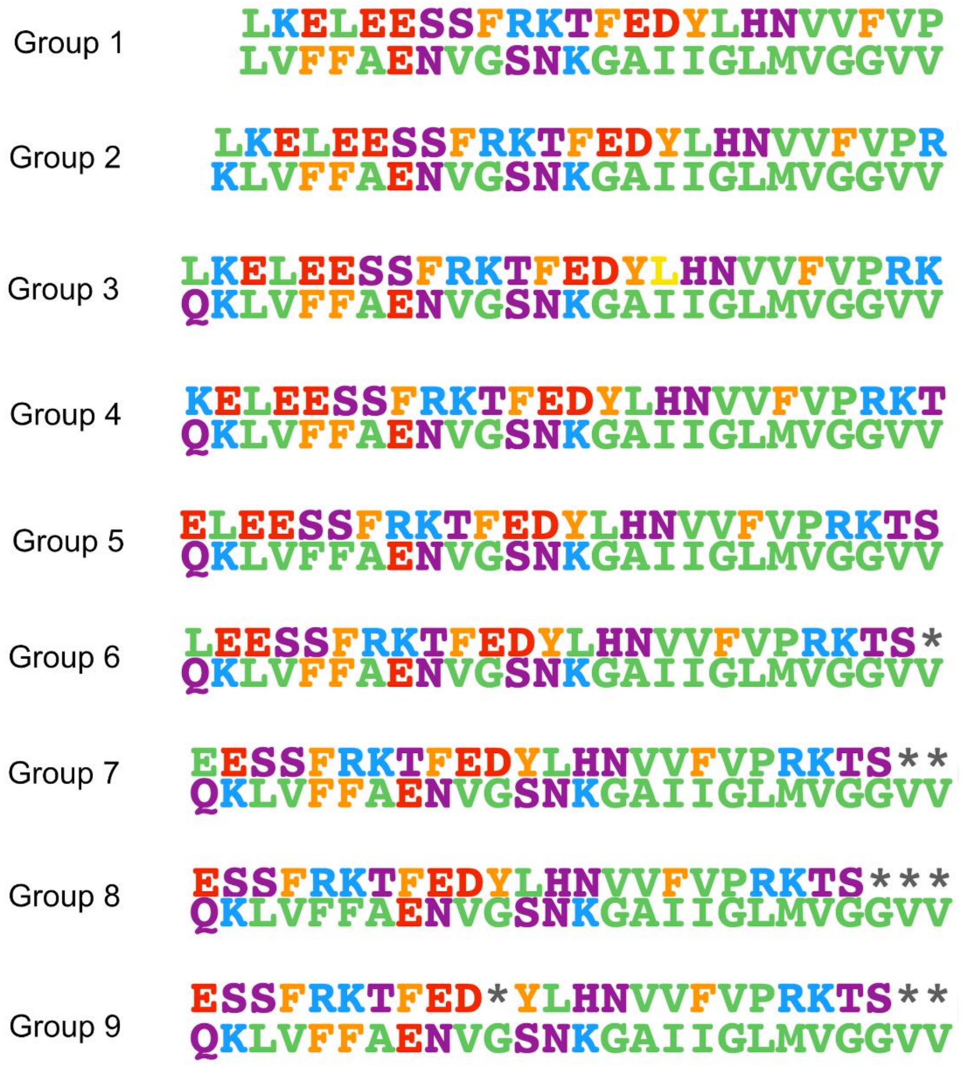

Supplementary Figure 1 Threading approach of the predicted sequence of aggregation of IRct. Pairwise alignments between IRct (top sequence) and oA$\beta_{1\text{-}40}$ subunit sequence (bottom sequence). Groups show different pairwise alignments. Legend of residues color is indicated at the bottom of the image. Hydrophobic amino acids are shown in green, polar residues in purple, aromatic residues in orange, negatively charged residues in blue and positively charged residues in red. Asterisks are gaps in the alignments.

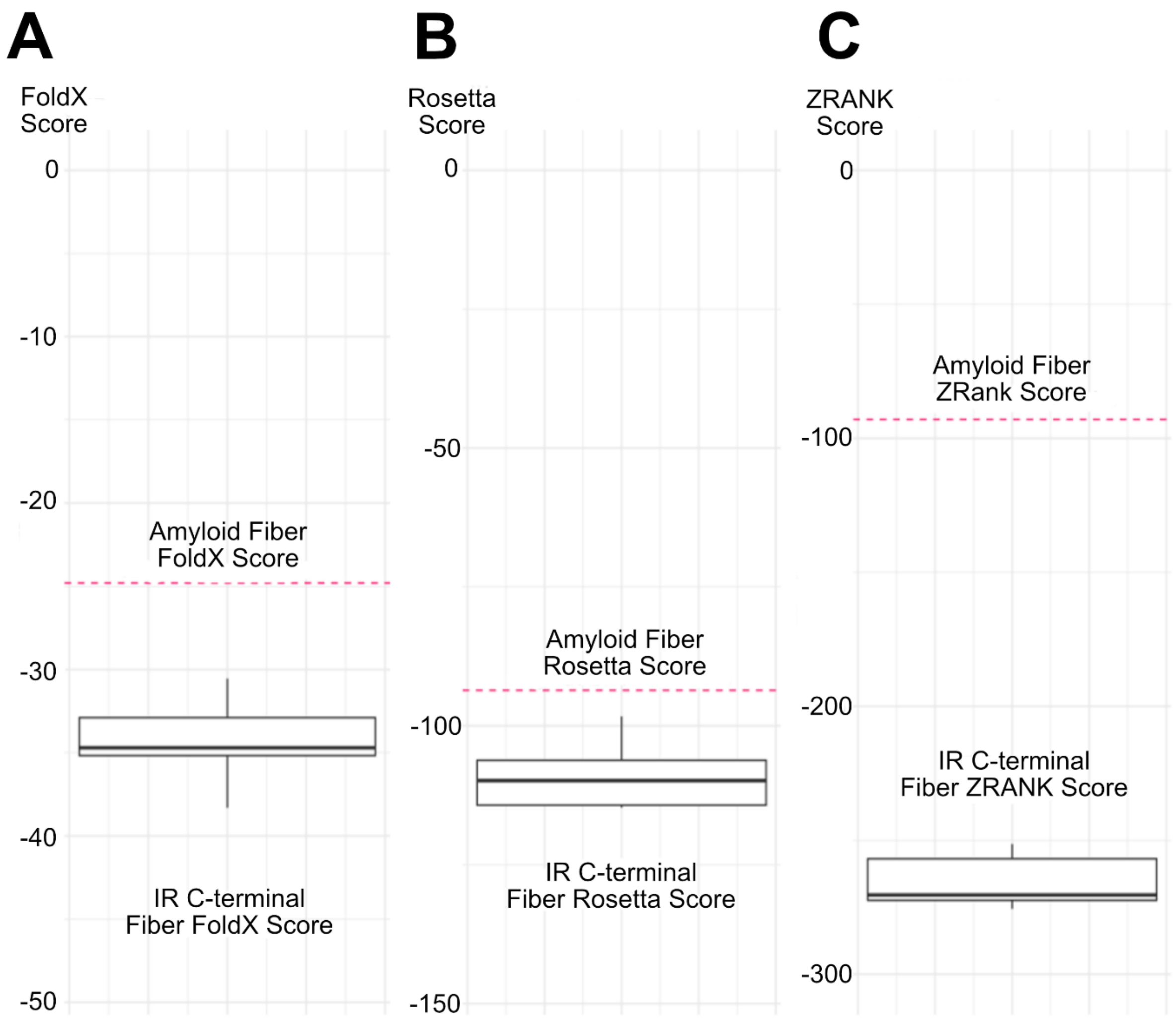

Supplementary Figure 2 Model of oA$\beta_{1\text{-}40}$ binds to IR with a similar energy to insulin. Distribution of binding energies calculated with (**A**) FoldX, (**B**) Rosetta Package (program InterfaceAnalyzer), and (**C**) ZRank for the energy of the models of IRct-oA$\beta_{1\text{-}40}$ (boxplot) against the native oA$\beta_{1\text{-}40}$ structure scores for each program (pink line). Lower values mean more stable.

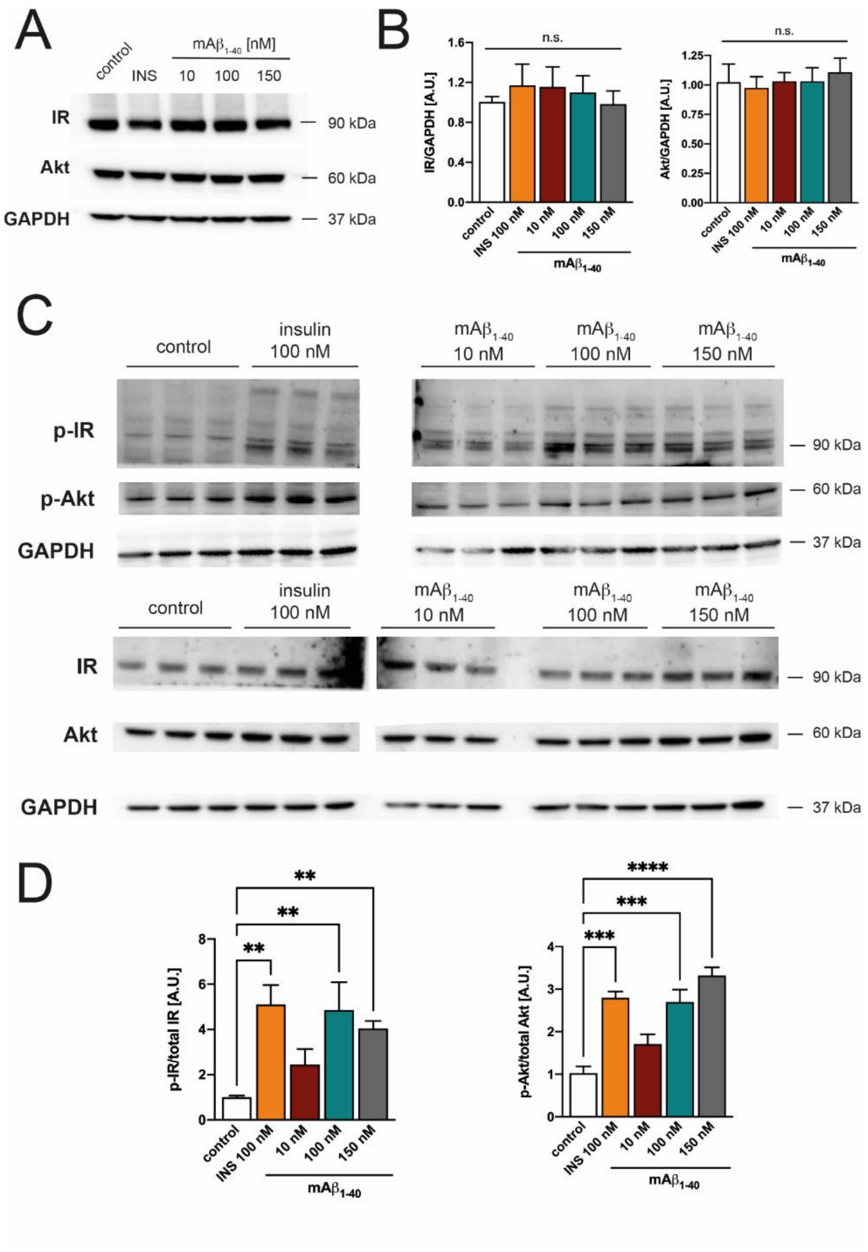

Supplementary Figure 3. Treatments do not induce changes on IR or Akt total levels. (**A**) Human neuroblastoma cells were treated with 10 nM, 100 nM, 150 nM m$A\beta_{1-40}$, or 100 nM of insulin for 10 min. Samples were extracted as indicated in the M&M section and WB were performed using anti-IR, anti-Thr308-Akt and anti-GAPDH. A representative WB is shown. (**B**) Quantification of 3 independent experiments performed by WB as indicated in (A). Levels of total IR (left) and total Akt (right) were nomarlised to GAPDH. n.s. compared to untreated controls by one-way ANOVA plus Student-Newman-Keuls as post hoc test. (**C**) Cells were treated as in (**A**) and WB were performed with anti-p-IR, anti-IR, anti-p-Thr308-Akt, anti-Akt and anti-GAPDH. (**D**) Quantification p-IR (left) and p-Thr308-Akt, (right) normalized to the total levels of IR or Akt, and to GAPDH. Data are the mean ± SEM of the 3 independent experiments showed in (**C**). ***p* <0.01, *** *p* < 0.001 **** *p* < 0.0001 by one-way ANOVA plus Student-Newman-Keuls as post hoc test.

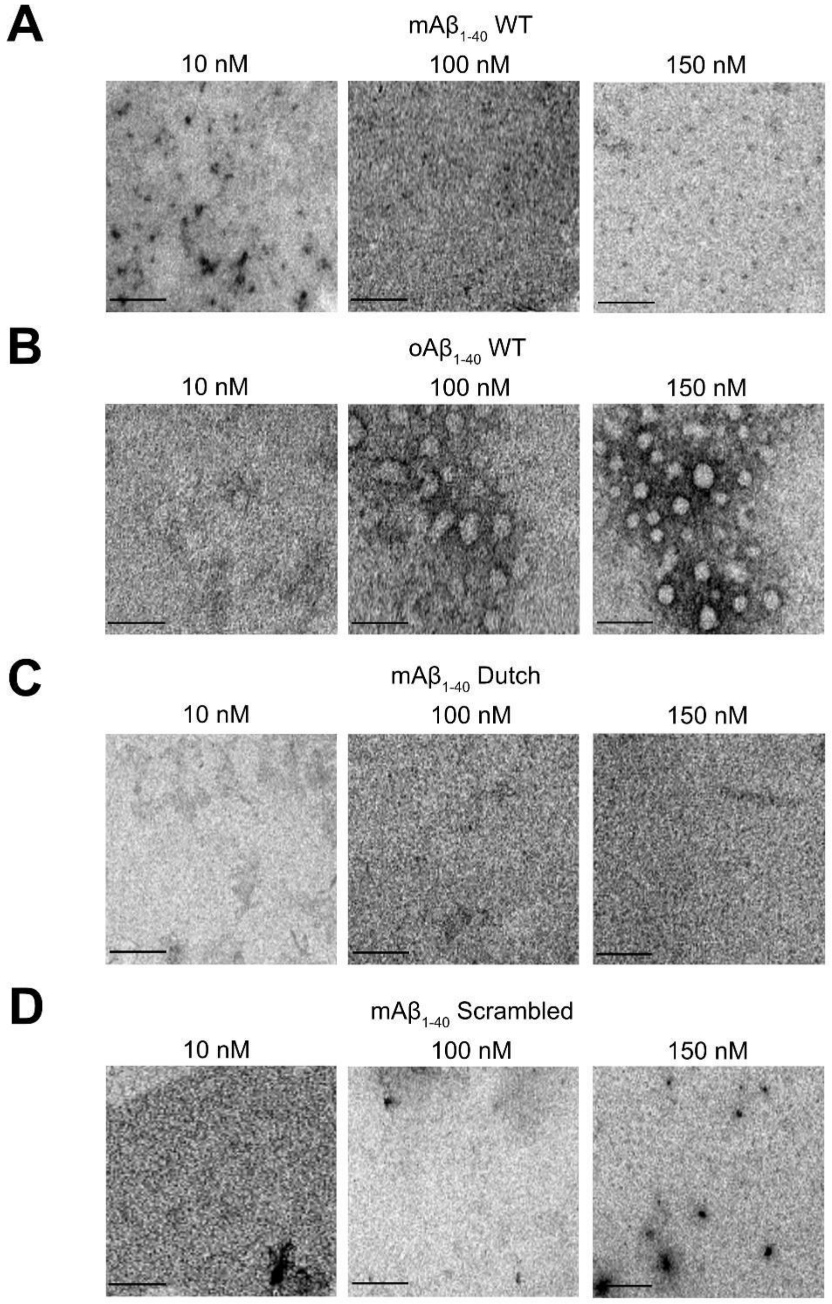

Supplementary Figure 4 Aβ_{1-40} structural analysis by TEM. Representative images obtained by TEM of (**A**) mAβ_{1-40} wild type, (**B**) oAβ_{1-40} wild type, (**C**) mAβ_{1-40} Dutch and (**D**) mAβ_{1-40} Scrambled. Amyloid structures as oligomers were only observed in the samples from oAβ_{1-40} wild type (**B**) while the other mAβ_{1-40} samples did not show amyloid structures. Bars represent 125 nm.

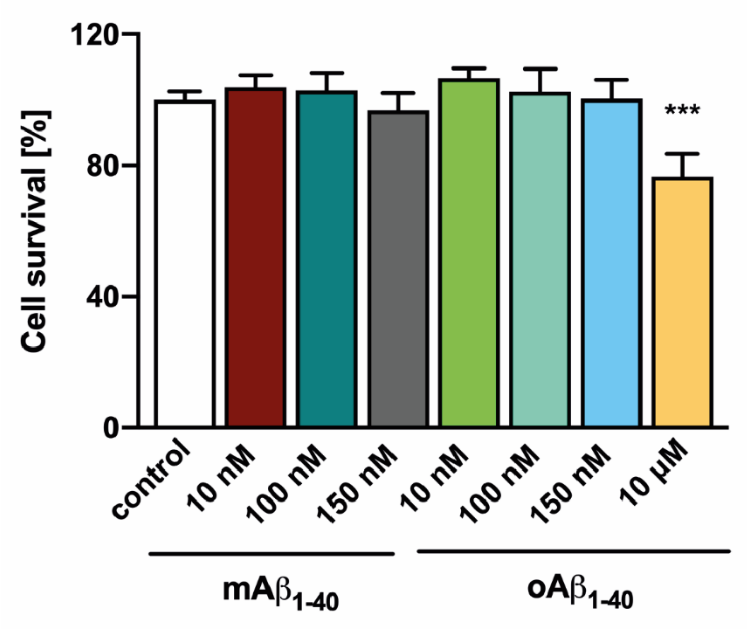

Supplementary Figure 5 Nanomolar concentrations of monomeric or oligomeric A$\beta_{1\text{-}40}$ do not induce cell death. Human neuroblastoma cells were treated during 24 h with increasing concentrations of mA$\beta_{1\text{-}40}$ (10 nM, 100 nM, and 150 nM) or oA$\beta_{1\text{-}40}$ (10 nM, 100 nM, 150 nM, and 10 µM). Cell survival was analysed by MTT reduction assay. 10 µM oA$\beta_{1\text{-}40}$ was used as a positive control since it is well known that it is toxic at this concentration. Data are the mean ± SEM of 3 independent experiments. *** $p < 0.001$ by one-way ANOVA plus Student-Newman-Keuls as post hoc test.

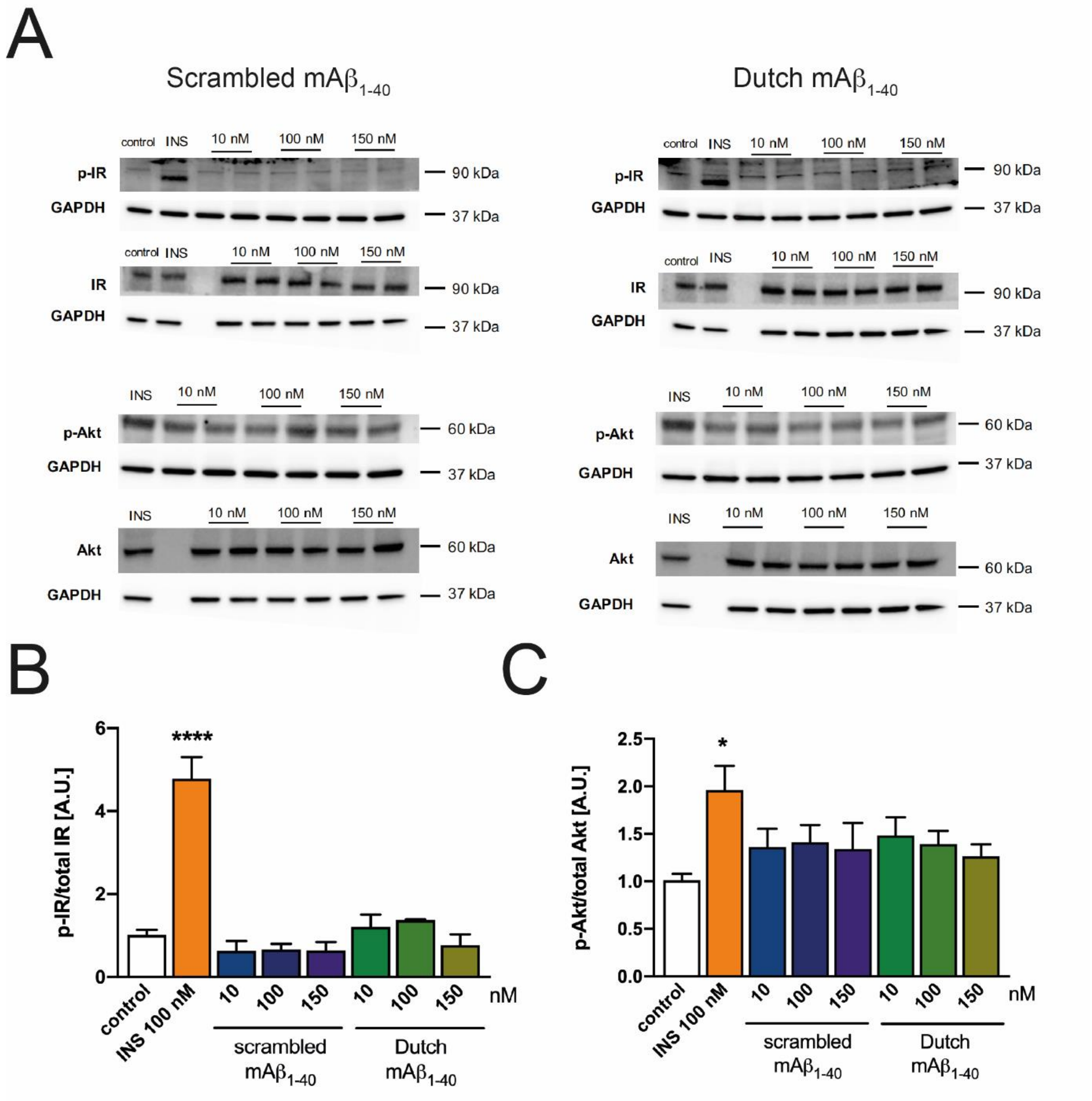

Supplementary Figure 6 mAβ1-40 Scrambled and Dutch do not induce the phosphorylation of IR nor Akt. (**A**) Human neuroblastoma cells were treated with 10 nM, 100 nM, 150 nM mAβ1-40 Scrambled and Dutch or 100 nM of insulin for 10 min. Samples were extracted as indicated in the M&M section and WB were performed using anti-p-IR, anti-IR, anti- p-Thr308-Akt, anti-Akt and anti-GAPDH. A representative WB is shown. (**B**, **C**) Band quantifications of p-IR regarding total IR (**B**) and p-Thr308-Akt regarding total Akt (**C**). Data are the mean ± SEM of the 3 independent experiments. * p <0.01, **** p < 0.0001 by one-way ANOVA plus Student-Newman-Keuls as post hoc test.

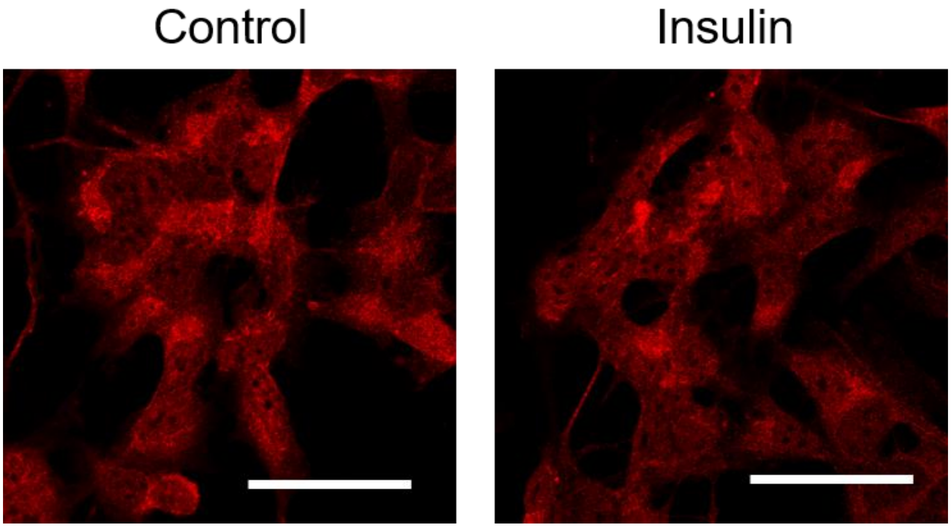

Supplementary Figure 7 Insulin does not induce GLUT4 translation. Cells were treated for 10 min with 100 nM insulin afterwards cells were permeabilized and fixed. Total expressed GLUT4 (intracellular and extracellular) was labelled with an anti-GLUT4 Ab (red). Same level of GLUT4 staining was obtained in treated and untreated cells. Bars represent 20 nm.

A

$$\mathrm{IR} + \mathrm{Ins} \underset{k_{i-}}{\overset{k_{i+}}{\rightleftharpoons}} \mathrm{IR{\cdot}Ins} \xrightarrow{k_{id}} \mathrm{S}\,, \qquad \{1\}$$

$$\mathrm{IR} + \mathrm{mA}\beta \underset{k_{m-}}{\overset{k_{m+}}{\rightleftharpoons}} \mathrm{IR{\cdot}mA}\beta \xrightarrow{k_{md}} \mathrm{S}\,, \qquad \{2\}$$

$$\mathrm{IR} + \mathrm{oA}\beta \underset{k_{a-}}{\overset{k_{a+}}{\rightleftharpoons}} \mathrm{IR{\cdot}oA}\beta \xrightarrow{k_{ad}} \varnothing\,, \qquad \{3\}$$

$$\mathrm{mA}\beta + \mathrm{oA}\beta \underset{k_{u}}{\overset{k_{b}}{\rightleftharpoons}} \mathrm{oA}\beta\,, \qquad \{4\}$$

$$\varnothing \xrightarrow{\alpha_m} \mathrm{mA}\beta\,, \qquad \{5\}$$

$$\varnothing \xrightarrow{\alpha_r} \mathrm{IR} \qquad \{6\}$$

$$\varnothing \xrightarrow{\alpha_i} \mathrm{Ins} \qquad \{7\}$$

B

$$\frac{dR}{dt} = \alpha_r - k_i I R - k_m M R - k_a A R - \delta_r R \qquad \text{(S1)}$$

$$\frac{dI}{dt} = \alpha_i - k_i I R \qquad \text{(S2)}$$

$$\frac{dM}{dt} = \alpha_m - k_m M R - k_b M A + k_u A \qquad \text{(S3)}$$

$$\frac{dA}{dt} = k_b M A - k_u A - k_a A R \qquad \text{(S4)}$$

$$k_i = \frac{k_{id}k_{i+}}{k_{i-} + k_{id}}, \qquad k_m = \frac{k_{md}k_{m+}}{k_{m-} + k_{md}}, \qquad k_a = \frac{k_{ad}k_{a+}}{k_{a-} + k_{ad}} \qquad \text{(S5)}$$

$$S = k_i I R + k_m M R \qquad \text{(S6)}$$

$$R_{\mathrm{st}} = \frac{\alpha_r - \alpha_i - \alpha_m}{\delta_r}, \qquad I_{\mathrm{st}} = \frac{\alpha_i \delta_r}{k_i(\alpha_r - \alpha_i - \alpha_m)} \qquad \text{(S7)}$$

$$A_{\mathrm{st}} = 0, \qquad M_{\mathrm{st}} = \frac{\alpha_m}{k_m R_{\mathrm{st}}} \qquad \text{(S8)}$$

$$A_{\mathrm{st}} = \frac{k_b \alpha_m - k_u k_m R_{\mathrm{st}} - k_a k_m R_{\mathrm{st}}^2}{k_b k_a R_{\mathrm{st}}}, \qquad M_{\mathrm{st}} = \frac{\alpha_m - k_a A_{\mathrm{st}} R_{\mathrm{st}}}{k_m R_{\mathrm{st}}} \qquad \text{(S9)}$$

Supplementary Figure 8 Kinetic reactions of the interaction of insulin, mAβ$_{1\text{-}40}$ and oAβ$_{1\text{-}40}$. (A) Biochemical reactions of the interactions of insulin, mAβ$_{1\text{-}40}$ and oAβ$_{1\text{-}40}$. S stands for "signaling", and we assume that neither the receptor nor any of its ligands are recycled after endocytosis. (**B**) Differential equations for the concentrations of the free species: insulin (I), mAβ$_{1\text{-}40}$ (M), oAβ$_{1\text{-}40}$ (A) and the IR itself (R). The δ_r represents the spontaneous rate of endocytosis of the IR, and we have defined the effective binding rates showed in Eqs. (S5). Finally, the signaling activity S produced by reactions {1} and {2} can be computed to be as showed in Eq. (S6). We now look for the steady-state solutions of system (S1)-(S4). To that end we set all left-hand-side derivatives to 0 and substract Eqs. (S3)-(S4) from Eq. (S1). This leads to an expression involving only I and R, which can be solved together with the steady-state expression of Eq. (S2), leading to analytic expressions for the steady state concentrations of IR and insulin as a function of the system parameters showed in Eqs. (S7). Finally, we can use these two expressions in Eqs. (S3)-(S4) to obtain the steady-state concentrations of mAβ$_{1\text{-}40}$ and oAβ$_{1\text{-}40}$. There are two different solutions showed in Eqs. (S8) and (S9).

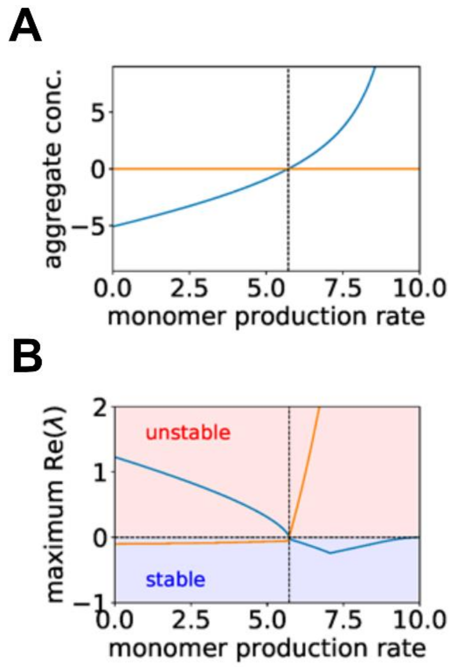

Supplementary Figure 9 Transcritical bifurcation as the monomer production rate α_m increases. (**A**) Steady-state (equilibrium) value of the oA$\beta_{1\text{-}40}$ concentration as a function of α_m. The trivial solution A = 0 is shown in orange, and the nontrivial solution is shown in blue. (**B**) Maximum real part of the eigenvalues governing the stability of the two solutions. When this maximum real part is positive (negative) the equilibrium is unstable (stable). Together, the two plots show that the only stable equilibrium for each value of α_m is that in which A is larger than or equal to 0 (the orange solution at the left of the vertical dashed line, and the blue solution at the right of that line). The parameter values are those given in Supplementary Table 2.

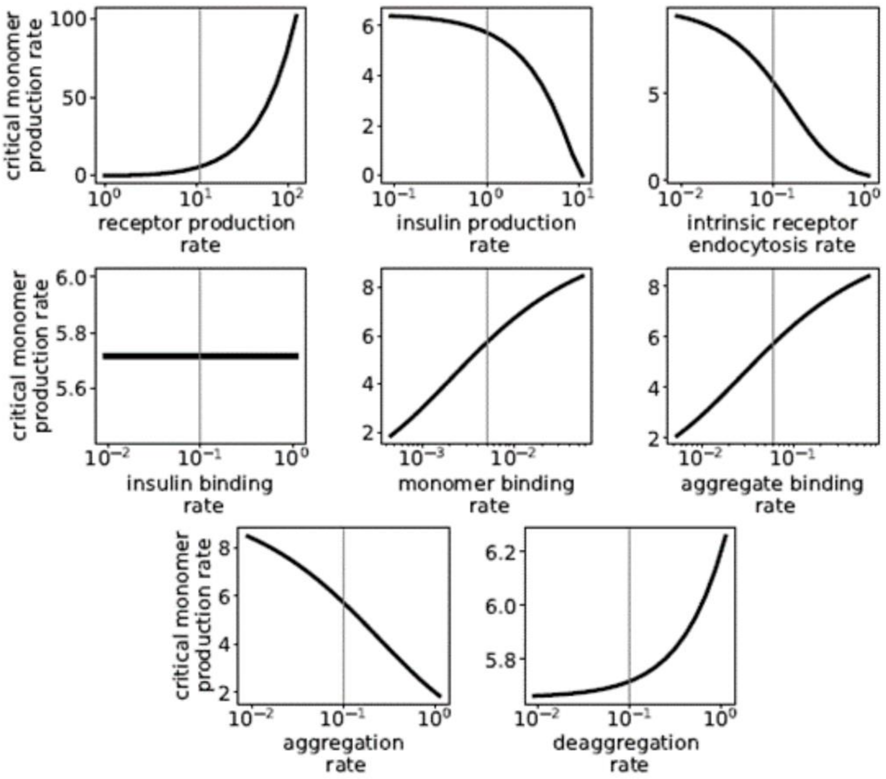

Supplementary Figure 10 The monomer production rate is a critical value. Dependence of the critical value of the monomer production rate, α_m, at which the transcritical bifurcation occurs, on all other parameters of the system. In each plot, only one parameter is varied, as denoted by the x-axis label, while the other parameter values are fixed to those of Supplementary Table 2. The vertical gray line in each plot represents the baseline value of the parameter being varied, as given in Supplementary Table 2.

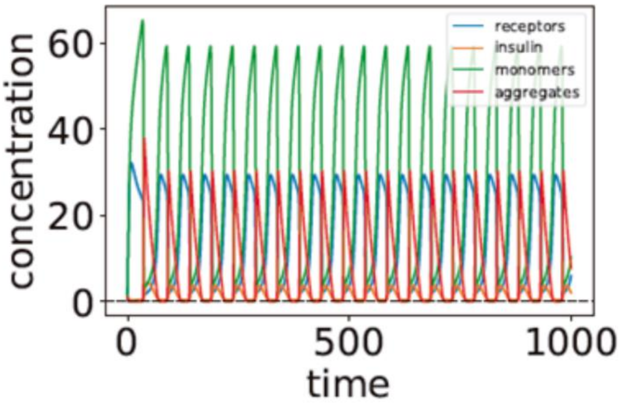

Supplementary Figure 11 Oscillatory dynamics of the model. Assumed when the binding rate of the aggregate to the insulin receptor is increase three-fold with respect to the baseline parameters given in Supplementary Table 2.

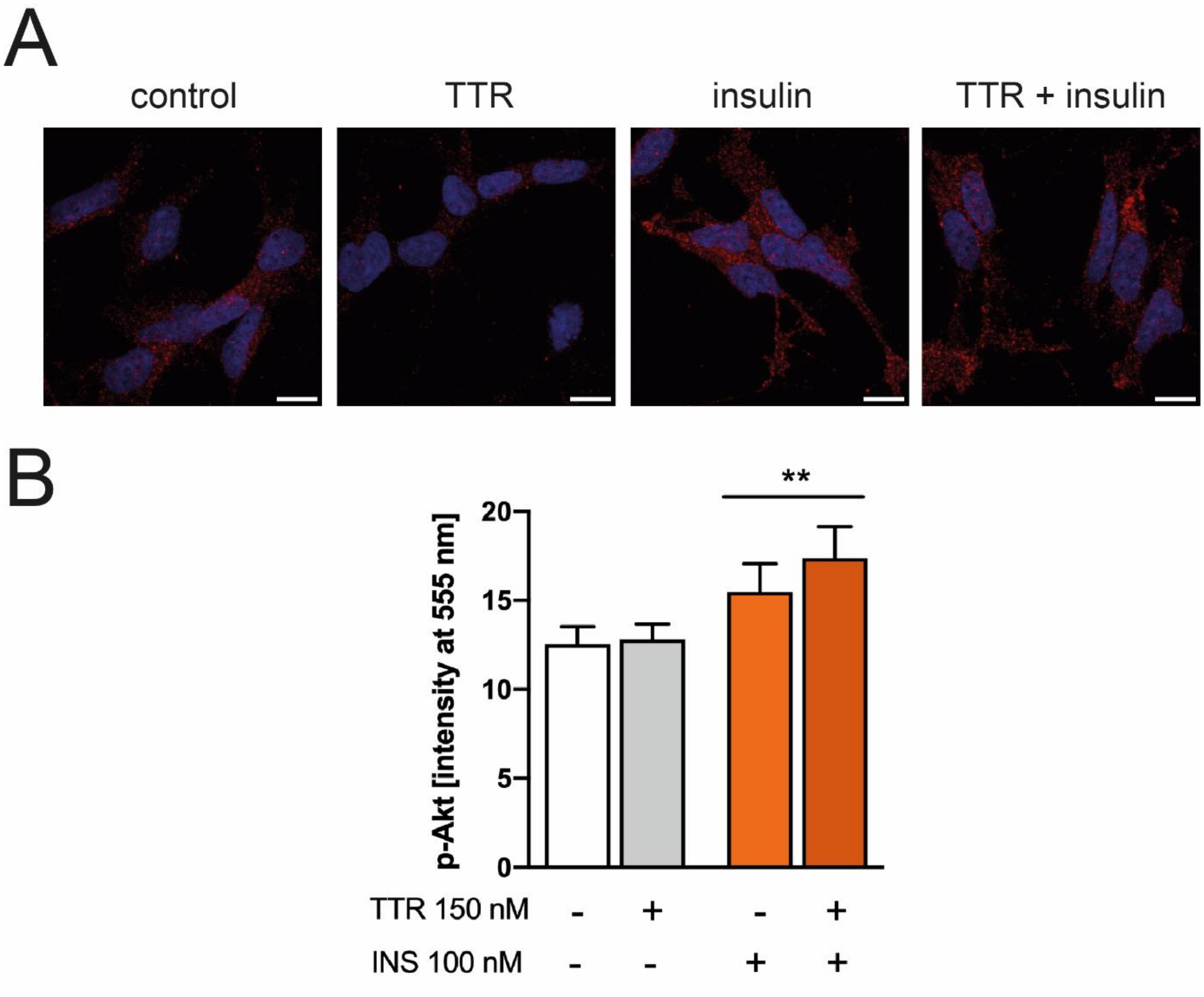

Supplementary Figure 12 TTR oligomers do not block insulin signalling. (A) Human neuroblastoma cells were treated with 150 nM oligomeric TTR for 30 min and then in the presence/absence of 100 nM insulin for 10 mins. Representative immunofluorescence images were obtained labelling p-Akt (red) and nuclei with DAPI (blue). Bars represent 10 nm. (**B**) Quantification of the inmunoflorescence results obtained as performed in (A). Data are the mean ± SEM of 3 independent experiments. ** $p < 0.01$ by one-way ANOVA plus Student-Newman-Keuls as post hoc test.

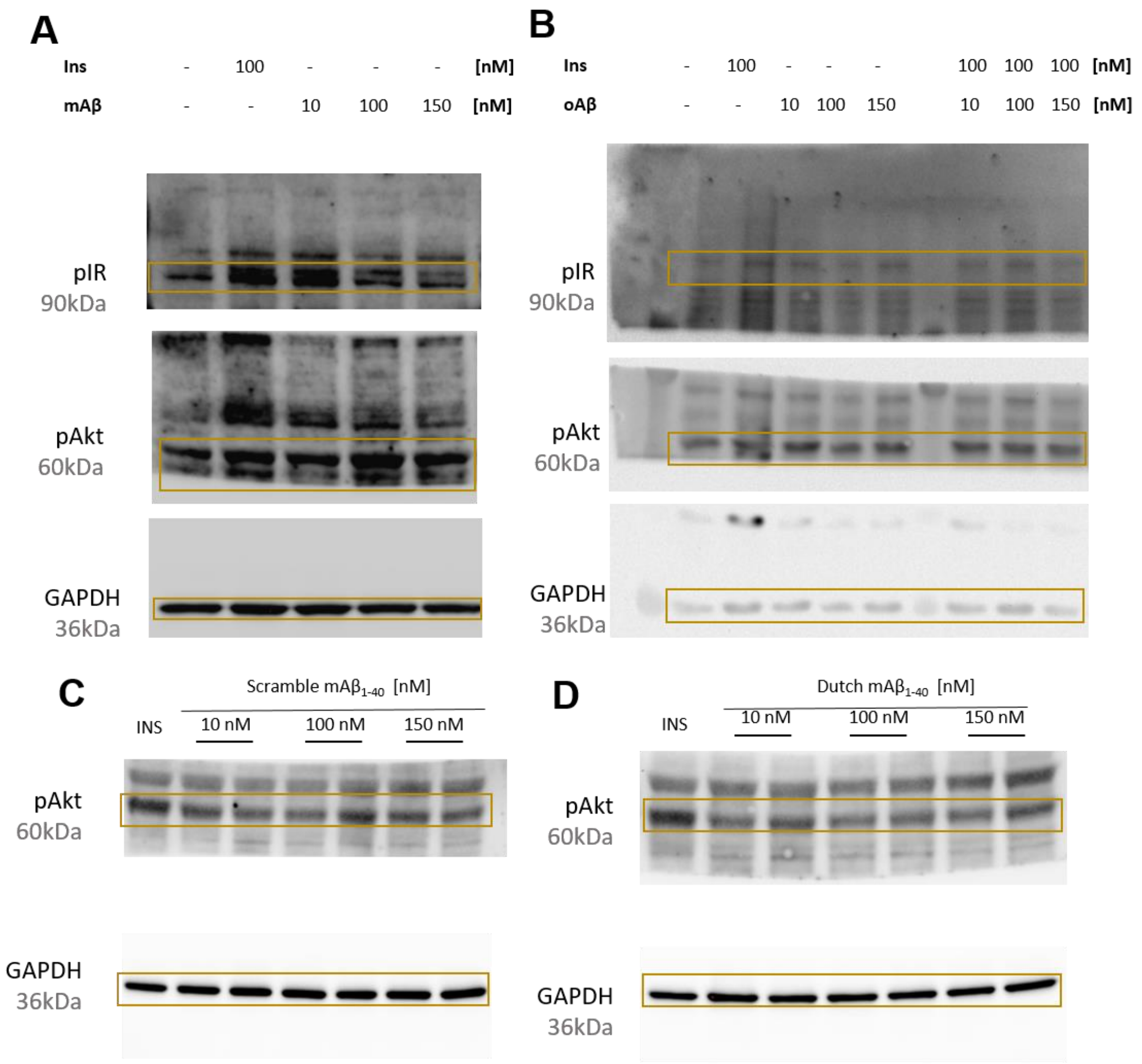

Supplementary Figure 13 Uncropped Western Blot showed in the figures of the article. (**A**) WB of Fig. 3A. (**B**) WB of Fig. 8A. (**C-D**) WB of Supplementary Figure 6A.
